# Terpenoids as Potential Geroprotectors

**DOI:** 10.3390/antiox9060529

**Published:** 2020-06-17

**Authors:** Ekaterina Proshkina, Sergey Plyusnin, Tatyana Babak, Ekaterina Lashmanova, Faniya Maganova, Liubov Koval, Elena Platonova, Mikhail Shaposhnikov, Alexey Moskalev

**Affiliations:** 1 Laboratory of Geroprotective and Radioprotective Technologies, Institute of Biology, Komi Science Centre, Ural Branch, Russian Academy of Sciences, 28 Kommunisticheskaya st., 167982 Syktyvkar, Russia; kateplus@mail.ru (E.P.); sergius-plusnin@yandex.ru (S.P.); yanat-b@mail.ru (T.B.); ekaterinalashmanova@yandex.ru (E.L.); lyubov.schilova@yandex.ru (L.K.); platonova.e.u@ib.komisc.ru (E.P.); shaposhnikov@ib.komisc.ru (M.S.); 2 Pitirim Sorokin Syktyvkar State University, 55 Oktyabrsky Prosp., 167001 Syktyvkar, Russia; 3 Initium-Pharm, LTD, 142000 Moscow, Russia; fmaganova@gmail.com

**Keywords:** terpenoids, carotenoids, antioxidants, aging, lifespan, geroprotectors

## Abstract

Terpenes and terpenoids are the largest groups of plant secondary metabolites. However, unlike polyphenols, they are rarely associated with geroprotective properties. Here we evaluated the conformity of the biological effects of terpenoids with the criteria of geroprotectors, including primary criteria (lifespan-extending effects in model organisms, improvement of aging biomarkers, low toxicity, minimal adverse effects, improvement of the quality of life) and secondary criteria (evolutionarily conserved mechanisms of action, reproducibility of the effects on different models, prevention of age-associated diseases, increasing of stress-resistance). The number of substances that demonstrate the greatest compliance with both primary and secondary criteria of geroprotectors were found among different classes of terpenoids. Thus, terpenoids are an underestimated source of potential geroprotectors that can effectively influence the mechanisms of aging and age-related diseases.

## 1. Introduction

Terpenoids are the largest group of plant secondary metabolites [1]. There are tens of thousands of naturally occurring hydrocarbons and they are one of the most structurally diverse classes of natural compounds. Terpenoids consist of C = 5, 10, 15, 20…, *n* > 40 carbon units and are classified as hemiterpenes (C5), monoterpenes (C10), sesquiterpenes (C15), diterpenes (C20), triterpenes (C30), tetraterpenes or carotenoids (C40), and polyterpenes (Cn, *n* > 40) [1]. Extensive biological investigations revealed a wide range of pharmacological and physiological activities of terpenoids and their derivatives [2,3,4]. However, terpenoids are rarely associated with anti-aging properties and may be underestimated as potential geroprotectors.

Geroprotectors are the pharmacological agents that decrease the rate of aging and extend lifespan. Despite the fact that terpenoids are the broad class of compounds, only a few of its representatives are well-known geroprotectors [5]. However, they are attracting increasing interest and such a systematic review of geroprotectors of various classes of terpenoids is necessary.

We proposed a set of primary and secondary selection criteria for potential geroprotector [6]. Primary criteria that should be met:

1. The life extension in experiments with wild type animal models. The geroprotector should prolong the life of the model beyond the intact maximum lifespan, protecting it from one or more mechanisms of aging.

2. Improvement of molecular, cellular, and physiological biomarkers to a younger state or slow down the progression of age-related changes in humans.

3. Most potential geroprotectors are preventive only when applied at relatively high concentrations. The lifespan-extending dose should be several orders of magnitude less than the toxic dose.

4. Minimal side effects at the therapeutic dosage at chronic application.

5. The potential benefit of taking a geroprotector may come after a long period. Potential geroprotectors should initially improve some parameters of health-related quality of life: physical, mental, emotional, or social functioning of the person.

Secondary selection criteria for potential geroprotector:

6. The target or mechanism of action of the geroprotector that extends the lifespan of the model should be evolutionarily conserved.

7. Reproducibility of geroprotective effects on different model organisms increases the possibility that effects will also be discovered in humans, even in the absence of a known conserved target.

8. Candidate geroprotectors should be able to delay the progress of one or several age-associated diseases in humans.

9. Potential geroprotectors should increase organism resistance to unfavorable environmental factors.

This review discusses terpenoid compounds belonging to different classes of this large group of substances, in terms of meeting the criteria for potential geroprotector and their potential for clinical use in relation to age-dependent diseases.

## 2. Extraction and Analysis of Terpenoids

Detection and structural identification play an important role in elucidating potential activities and developing therapeutic approaches to natural geroprotectors. Currently, advancement has been made in the determination of structure and studying of the chemical features of terpenes and terpenoids, as well as methods for their extraction. These methods are constantly improving, and new approaches are being developed [7].

All terpenes and terpenoids contain a hydrocarbon skeleton, which is formed from five-carbon precursors (isopentenyl diphosphate and dimethylallyl diphosphate) and polymerizes with the formation of prenyl diphosphates of various lengths. As a result of the removal of the diphosphate group, the intermediate allyl cations can be subjected to a series of chemical cascades with the formation of various compounds with linear and/or cyclized hydrocarbon chains, which are then further modified by the addition of various functional groups and adducts [2,3,4]. This feature of terpenes and terpenoids creates their remarkable chemical diversity and requires an appropriate approach for extraction, detection, and purification [8,9,10].

The preparation and analysis of natural compounds include four stages: (1) the release of biologically active compounds; (2) extraction; (3) purification of a target substance from an extract; (4) identification of the chemical structure of a target compound. The use of specific methods is determined depending on the size and complexity of a molecule, its physical properties (polarity, volatility), chemical properties, and some other parameters [7]. The polarity of a molecule is the most important feature that should be considered when determining the method of extraction, analysis, and purification of a substance [8,9,11].

Most primary terpenes without chemical modifications are non-polar. Some of them with the smallest molar mass (especially mono- and sesquiterpenes) can be volatile [12]. Non-volatile non-polar terpenes are extracted with hexane or other non-polar solvents [9]. In addition, to extract this fraction, silica can be used as a stationary phase. For the analysis of the obtained samples of non-polar terpenes, purification of the target molecules, and their structural identification, gas chromatography is used [9]. Additionally, thin layer chromatography (for more accurate identification of specific fractions with the target molecule) and high-performance liquid chromatography (for thorough purification) are applicable [7].

Isolation and purification of volatile non-polar terpenes have limitations associated with the need for their capture and the difficulties of separating substances from each other [7,8]. There is a molecular capture technique and novel approaches, such as solid-phase microextraction [13,14] or microwave-assisted extraction [15]. For structural identification, gas chromatography is also used [12].

The process of extraction and analysis of compounds with modifications requires other methods because they are polar molecules with greater variability of chemical properties. The degree of polarity depends on the type of modifications and their quantity. Modifications by the addition of methyl or hydroxyl groups provide a relatively low polarity of the compounds. For extraction, a suitable approach is the use of hexane (or polar methanol) as a solvent, but for analysis, liquid chromatography. However, it is possible to use gas chromatography, supplemented by derivatization, as well as thin-layer chromatography [7].

Modifications such as acylation, aroylation, glycosylation, and the addition of other functional groups increase the size and polarity of triterpenoids. For the extraction of such fractions, it is recommended to use polar solvents, in particular, methanol (or alternative methods, such as extraction with ionic liquid), and for analysis, high-performance liquid chromatography/electrospray tandem mass spectrometry [16,17]. Additional methods may be required to prepare the samples, depending on the chemical structure of specific terpenes and terpenoids [7,11].

## 3. Terpenoids as Potential Geroprotectors

### 3.1. Monoterpenes

**Figure 1:**
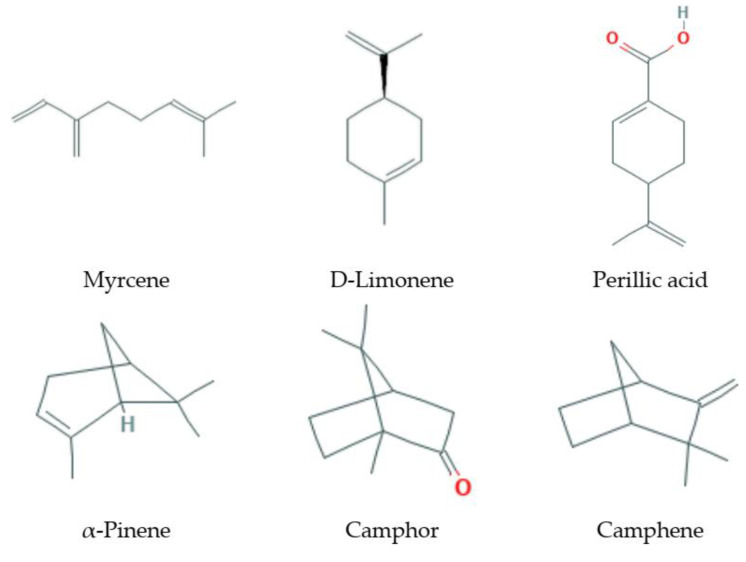
Some monoterpenes that increase lifespan and delay age-related diseases.

Monoterpenes (Figure 1) are isoprene dimers; they have the composition C_10_H_16_. These are easily volatile compounds with a pleasant smell that form the basis of essential oils of plants. According to the structure, monoterpenes are divided into two large groups: acyclic, with an open carbon chain (for example, myrcene, ocimene) and cyclic, which can contain both one cycle (limonene) and several (fennenes, pinenes); some bicyclic monoterpenes contain cyclopropane cycles (sabinene).

#### 3.1.1. Natural Sources

These substances are the main components of essential oils of fruits and the volatile turpentine fraction of conifer oleoresins. Camphene is found in the juniper oil, pettigrein—in the pine oil; dipentene—in oils of bergamot, coriander, sweet dill, lemon; limonene—in oils of bergamot, cumin, carrot, sweet dill, lemon, neroli, orange; pinene—in oils of coriander, cypress, eucalyptus, sweet dill, pine, rosemary; sylvestren—in oils of cypress, pine, and many other tree oils. Monoterpenes myrcene and ocimene are contained in essential oils of hops and laurel. Monoterpene alcohols, such as geraniol, are the main components of essential oils of rose, geranium, and other flower essences. Aldehydes (geranial) have a citrus smell and are contained in lemon essential oils. Camphor, a bicyclic monoterpene, is one of the major constituents of essential oils from rosemary, lavender, and sage [18]. In different parts of pine, the following were found: γ-terpinene and β-pinene—in the needles; limonene—in the bark; α-pinene and limonene—in the pollen [19]. Monoterpenes limonene is contained in the lemon oil and turpentine, and is a part of the oil of cumin. The highest content of limonene was found in representatives of the genus *Citrus* (*Rutaceae*) [20,21].

#### 3.1.2. Lifespan Extension on Different Models

The hormetic-like effect of limonene was found in the Mediterranean fruit fly (*Ceratitis capitata*) model: despite the frank toxicity of limonene in high doses (LD_90_—39.74 nL per male and 75.51 nL per female), low doses (LD_20_—3.47 nL per male and 12.26 nL per female) increased the lifespan and female fertility in the case of protein-free diet [22]. Small concentrations of limonene (0.011 and 0.046 mM) also significantly increase both average and maximum lifespan of *Drosophila melanogaster* [23]. Exposure of the olive fruit flies (*Bactrocera oleae*) by the aroma of α-pinene, which is present in both olive fruit and leaves, increased the lifespan in males and fecundity in females under dietary restricted conditions [24]. However, studies conducted on the Mediterranean fruit fly did not confirm the pro-longevity effect [22].

#### 3.1.3. Effects on Stress-Resistance

Low doses of camphor could be beneficial, inducing neurohormesis [25] or anti-tumor activity [26]. Camphor as a component of cosmetics can delay skin aging, decreasing the activity of elastase, elevating collagen expressions, activating proliferation of human primary dermal fibroblasts, and attenuating cellular senescence [18]. It has anti-mutagenic effects in small doses [27]. α-Pinene attenuated UV-induced photoaging by inhibiting the expression of matrix metalloproteinases in mouse skin [28]. Antioxidant properties were found for menthol [29] and γ-terpinene [30], these compounds prevent peroxidation of lipids and fatty acids. In *Caenorhabditis elegans,* the antioxidant activity of the mint essential oil was comparable to ascorbic acid [29]. Perillic acid showed protective properties in radiation-induced oxidative stress [31]. A mixture of geraniol and camphene prevented mitochondrial dysfunction, oxidative stress, and the release of apoptotic proteins in the liver during the nimesulide poisoning in rats [32]. The methanol extract of fennel seeds containing L-limonene softened oxidative stress and protected mouse cells from the damage caused by active forms of oxygen [33].

#### 3.1.4. Toxicity and Side Effects

High doses of camphor have pronounced toxicity. In the Ames test, monoterpenoids (camphor, 1,8-cineol, citral, citronellol, menthol, except for terpineol) showed no mutagenic properties [34]. Low doses of monoterpenes, such as camphor, eucalyptol, and thujone, have a cytoprotective and antimutagenic effect, however, in large doses, their effects are opposite [27]. The products of the interaction of limonene with oxygen (oxygen hydroperoxides) could be aromatic allergens [35].

#### 3.1.5. Life Quality Effects

Monoterpenes are often partially responsible for the aroma or odor of plants and are major odoriferous compounds of many flowers and fruits. Esters of terpene alcohols (citronellol, geraniol, nerol, farnesol, linalool, perillyl alcohol, menthol, borneol, carveol) are described as highly potent, reversible, and low toxic skin penetration enhancers [36]. D-limonene reduces overall stress levels and improves markers of inflammation [37,38,39]. D-limonene in Wistar rats caused the intense and persistent bradycardia associated with hypotension. In the in vivo model of arrhythmia, D-limonene (10 mg/kg) reduced the heart rate and arrhythmia [40]. In experiments with *Oreochromis niloticus*, *Citrus bergamia* peel oil containing limonene and linalool was added to the fish diet. Highest levels of total protein and lowest levels of serum cholesterol and triglycerides were observed in fish treated with 0.5 g per 100 g of bergamot oil, and fish growth rates were significantly increased [41].

#### 3.1.6. Suppression of Pro-Aging or Activation of Anti-Aging Molecular Targets or Pathways

Hormesis pathways activated by phytochemicals include NRF2 and FOXO transcription factors that stimulate the production of antioxidant enzymes, protein chaperones, and neurotrophic factors [25]. Camphor induced the proliferation of primary human skin fibroblasts via PI3K/AKT and ERK signaling pathways. It attenuated an increase of the β-galactosidase (SA-β-gal) activity associated with aging, induced the expression of collagen (IA, IIIA, IVA types) and elastin in primary human dermal fibroblasts [18]. Myrcene ameliorates human skin extrinsic aging via decreasing the production of ROS, MMP-1, MMP-3, and IL-6, and increasing of TGF-1 and type I procollagen secretions. Myrcene treatment reduces the induction of mitogen-activated protein kinase (MAPK)-related signaling molecules such as p-ERK, p-p38, and p-JNK, and AP-1 [42]. Abisil, a substance of terpenes of *Abies sibirica* enchances the activity of a cellular energy sensor—AMPK—in mice [43].

#### 3.1.7. Effects on Age-Related Diseases

Citronellol decreased hyperglycemia in streptozotocin-induced diabetic rats. The addition of citronellol to the STZ diet of rats positively influenced the maintenance of normal histological manifestation of liver cells and insulin-positive β-cells [44]. In a study of effects of limonene and perillic acid in C57BL/6 mice, a significant (65% and 67%) inhibition of the metastatic tumor formation was revealed [45]. Immunomodulatory activity (increase in total leukocyte count) was detected in Balb/c mice after the consumption of limonene and perillic acid [46]. D-limonene has chemopreventive activity against mammary, skin, liver, lung, and forestomach cancer in rodents [47]. D-limonene and its derivatives have chemotherapeutic and chemoprophylactic efficacy in cancer in various preclinical model systems [48]. On the cellular model of osteoarthritis, it was shown that myrcene has significant anti-inflammatory and anti-catabolic effects on human chondrocytes and is able to slow down the destruction of cartilage and the development of osteoarthritis. Myrcene and limonene prevent the increased expression of non-cartilage specific collagen I induced by IL-1β [49]. α-Terpineol has antitumor activity and acts by suppressing the transmission of NF-κB signals [50]. The protective effect of α-terpineol against disruption of synaptic plasticity of the hippocampus and spatial memory after transient cerebral ischemia in rats was revealed by facilitating long-term potentiation and suppressing lipid peroxidation in the hippocampus [51]. γ-Terpineol inhibited cell growth and caused apoptosis in human Bel-7402 cancer cells. A possible anti-cancer mechanism of γ-terpineol on human hepatoma cells is the induction of cellular apoptosis suppressing the growth of tumor cells [52]. Monoterpenes inhibit cell growth, cell cycle progression, and expression of the cyclin D1 gene in human breast cancer cell lines, and cause dose-dependent inhibition of cell proliferation [53]. Camphene reduces plasma cholesterol and triglycerides in rats with hyperlipidemia [54]. Terpenes of *Abies sibirica* affect molecular pathways associated with cancer and aging in human cells [5], induce apoptosis and inhibit proliferation in tumor cells in vitro, suppress tumor growth and angiogenesis in vivo [43].

#### 3.1.8. Additional Activities

The antibacterial activity of the essential oil of *Citrus hystrix* with a concentration of 2% (by weight) showed a strong inhibitory effect against *Bacillus subtilis* and *Escherichia coli* [21]. *Trichophyton rubrum* is a fungus that causes chronic dermatophytosis in humans. Geraniol and citronellol exhibit antimicrobial properties damaging cell wall and cell membrane of *T. rubrum* by inhibiting ergosterol biosynthesis [55]. The essential oil of *Santolina impressa*, which includes β-pinene, 1,8-cineole, limonene, camphor, has a fungicidal effect on *Cryptococcus neoformans, Epidermophyton floccosum*, and *Trichophytum rubrum* [56]. The essential oil of leaves of *Psidium guajava* inhibited pathogenic human bacteria *Curvularia lunata* [57]. Essential oils from various aerial parts of *Pinus eldarica* show antibacterial properties against *E. coli* (essential oil from pollen). Essential oil from the cortex inhibited the growth of *Candida albicans* and *Staphylococcus aureus*, as well as a decreased growth of *S. aureus*, under the influence of the essential oil from the needles [19].

### 3.2. Sesquiterpenes

**Figure 2:**
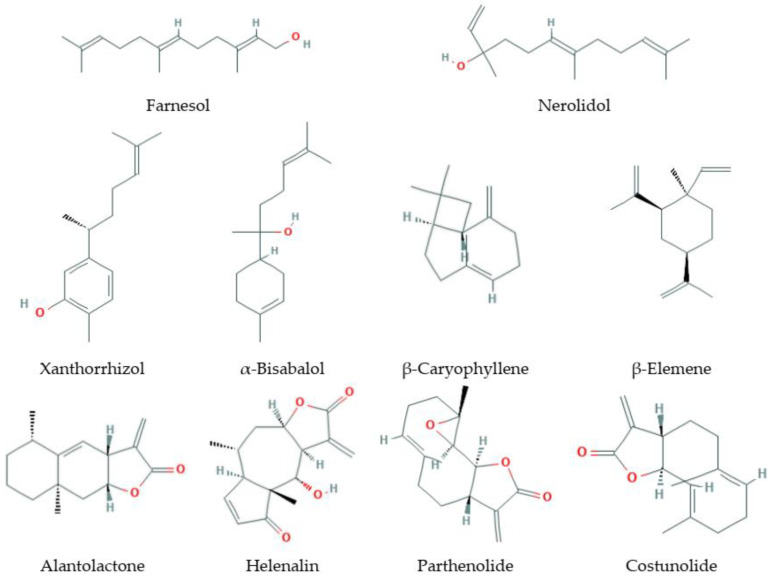
Some sesquiterpenes that increase lifespan and delay age-related diseases.

Sesquiterpenes (Figure 2) are C15-terpenoids built from three isoprene units. They are found particularly in higher plants and in many other living systems such as marine organisms and fungi. Usually sesquiterpenes are hydrocarbons or have oxygenated forms including lactones, alcohols, acids, aldehydes, and ketones.

#### 3.2.1. Natural Sources

Sesquiterpenes are a group of secondary metabolites in plants comprising a large group of over 5000 known compounds, being most common in families such as *Apiaceae, Araceae, Araliaceae, Asteraceae*, *Cactaceae, Euphorbiaceae, Lamiaceae, Rutaceae, Solanaceae*.

#### 3.2.2. Lifespan Extension on Different Models

Lifespan extension experiments were made only for β-caryophyllene. This compound possesses a wide range of biological activities including antioxidant, anti-inflammatory, anti-cancerous, and local anesthetic actions. Pant et al. (2014) used *C. elegans* as a model system to elucidate the stress modulatory and lifespan prolonging action of β-caryophyllene. It was found that a 50 μM dose of this sesquiterpene increased the lifespan of *C. elegans* by over 22% and significantly reduced intracellular free radical levels, maintaining cellular redox homeostasis [58].

#### 3.2.3. Effects on Stress-Resistance

It was shown that farnesol quells oxidative stress, reactive gliosis, and inflammation during acrylamide-induced neurotoxicity [59]. Nerolidol exhibits a protective effect against pentylenetetrazol-induced kindling, oxidative stress, and associated behavioral comorbidities in mice [60]. This compound has an effect against neuroinflammation, and oxidative stress induced by rotenone [61]. α-Bisabolol also has a protective effect on rotenone-induced toxicity in *D. melanogaster* [62], on altered hemodynamics, lipid peroxidation, and nonenzymatic antioxidants in isoproterenol-induced myocardial infarction [63]. It exhibits nephroprotective effects against ischemic-reperfusion acute kidney injury [64]. β-Elemene has a beneficial effect in traumatic brain injury by inflammatory pathway [65]. Xanthorrhizol prevents amyloid-β-induced oxidative modification and inactivation of neprilysin [66]. β-Caryophyllene has an antioxidant effect and plays a protective role for rat liver from CCl_4_-induced fibrosis by inhibiting hepatic stellate cell activation [67]. Furthermore, β-caryophyllene oxide has effects on xenobiotic-metabolizing enzymes in mice in vivo [68]. Costunolide protects mice against D-galactosamine and lipopolysaccharide-induced acute liver injury [69]. It exhibits antioxidant activity [70], for example, in experiment with H_2_O_2_-induced injury in PC12 cells [71]. In addition, this lactone protects against an ethanol-induced gastric ulcer in mice [72]. Parthenolide has a protective effect on LPS-induced acute lung injury [73]. Helenalin attenuates alcohol-induced hepatic fibrosis by enhancing ethanol metabolism, inhibiting oxidative stress, and suppressing HSC activation [74]. Alantolactone plays neuroprotective roles in traumatic brain injury in rats via anti-inflammatory, anti-oxidative, and anti-apoptosis pathways [75]. It prevents amyloid β25-35-induced toxicity in mouse cortical neurons and scopolamine-induced cognitive impairment in mice [76].

#### 3.2.4. Effects on Aging Biomarkers

β-Elemene decreased levels of plasma endotoxin, serum TNF-α, and hepatic CD14 expression in rats with liver fibrosis [77]. Xanthorrhizol has hypolipidemic activities [78]. β-Caryophyllene demonstrated a hypocholesterolemic effect in rats fed cholesterol and fat-enriched diet [79]. It reduced the atherogenic index and coronary risk index in hypercholesterolemic rats [80] and protected in vitro neurovascular unit against oxygen-glucose deprivation and reoxygenation-induced injury [81], also it made a significant reduction in intestinal lipofuscin levels [58].

#### 3.2.5. Toxicity and Side Effects

Nerolidol downregulates mitochondrial and cellular energetics [82]. It was also tested in in vivo genotoxicity assessment of nerolidol [83], demonstrating weak levels of dose-related DNA damage, and enhancing the average number of micronucleated cells. α-Bisabolol promotes cell death by inducing pores in mitochondria and lysosomes [84]. β-Caryophyllene showed the absence of adverse effects in female Swiss mice [85], and in a subchronic toxicity study in rats [86]. Costunolide induces micronuclei formation, chromosomal aberrations, cell cycle arrest, and mitochondrial-mediated apoptosis in Chinese hamster ovary cells [87]. Helenalin suppresses essential immune functions of activated CD4^+^ T-cells [88], Leydig, and adrenocortical cell steroidogenesis by inhibiting expression of the steroidogenic acute regulatory protein [89].

#### 3.2.6. Life Quality Effects

Farnesol exerts anti-inflammatory and anti-allergic effects on ovalbumin-sensitized and challenged asthmatic mice [90], ameliorates serum allergic antibody titers and lipid profiles [91], exerts an antinociceptive effect as confirmed by histopathological analysis of the striatum and hippocampus in mice [92]. β-Caryophyllene modulates feeding behavior [58] and has a therapeutic potential from several pharmacological points [93].

#### 3.2.7. Suppression of Pro-Aging or Activation of Anti-Aging Molecular Targets or Pathways

Farnesol and nerolidol can induce apoptosis of cancer cells [94,95]. β-Caryophyllene oxide potentiates TNF-α-induced apoptosis, and it inhibits invasion through down-modulation of NF-κB-regulated gene products [96]. β-Elemene enhances GAP-43 expression and neurite outgrowth by inhibiting RhoA kinase activation in rats with spinal cord injury [97]. It inhibits the proliferation of primary human airway granulation fibroblasts by downregulating the canonical Wnt/β-catenin pathway [98], attenuates macrophage activation and proinflammatory factor production via crosstalk with Wnt/β-catenin signaling pathway [99], induces the apoptosis of non-small-cell lung carcinoma cells through PERK/IRE1α/ATF6 pathway [100]. β-Elemene acts as an antitumor factor and downregulates the expression of survivin, Bcl-xL, and Mta-1 [101]. Xanthorrhizol induces apoptosis through ROS-mediated MAPK activation in human oral squamous cell carcinoma cells and inhibits DMBA-induced oral carcinogenesis in hamsters [102,103]. It was shown that xanthorrhizol regulates p53-, Bcl-2-, and caspase-dependent signaling pathway and induces apoptosis in HepG2 hepatoma cells [104]. β-Caryophyllene interacts with the main anti-aging genes SIR-2.1, SKN-1, and DAF-16 in *C. elegans* [58]. It attenuates focal cerebral ischemia-reperfusion injury via NRF2/HO-1 pathway in rats [105] and alleviates D-galactosamine and lipopolysaccharide-induced hepatic injury through suppression of the TLR4 and RAGE signaling pathways [106]. It exhibits antigenotoxic capacity in mice and has antioxidant and GST induction activities [107]. In addition, this compound ameliorates the Alzheimer-like phenotype in APP/PS1 mice through CB2 receptor activation and the PPARγ pathway [108].

Costunolide ameliorates lipoteichoic acid-induced acute lung injury via attenuating MAPK signaling pathway [109]. Activation of p53 by costunolide blocks glutaminolysis and inhibits proliferation in human colorectal cancer cells [110]. This compound protects lipopolysaccharide/D-galactosamine-induced acute liver injury in mice by inhibiting the NF-κB signaling pathway [111]. It inhibits proinflammatory cytokines and iNOS in activated murine BV2 microglia [112] and reveals inhibitory effects on the telomerase activity in human breast carcinoma cells [113]. Parthenolide is a direct inhibitor of the inflammasome [114]. It inhibits STAT3 signaling by covalently targeting Janus kinases [115], Wnt/β-catenin signaling by blocking synthesis of the transcriptional regulators TCF4/LEF1 [116] and FAK-mediated cell invasion [117]. Hsp72 is another intracellular target of this lactone [118]. Helenalin has differential effects on the proteome, metabolome, and the oxidative stress response in several immune cell types [119]. NF-κB p65 repression by helenalin contributes to the induction of autophagy cell death [120,121]. It produces inhibitory effects on 5-lipoxygenase and leukotriene C(4) synthase in human blood cells [122] and telomerase activity, attributed to the alkylation of the CYS445 residue [123]. Alantolactone induces apoptosis and suppresses migration in MCF-7 human breast cancer cells via the p38 MAPK, NF-κB, and NRF2 signaling pathways [124]. This lactone produces NRF2-mediated induction of detoxifying enzymes [125] and activation of apoptosis in human hepatoma cells [126]. Alantolactone exerts anti-inflammatory effects by inhibiting chemokine production and STAT1 phosphorylation in TNF-α and IFN-γ-induced in HaCaT cells [127] and sensitizes human pancreatic cancer cells to EGFR inhibitors through the inhibition of STAT3 signaling [128].

#### 3.2.8. Effects on Age-Related Diseases

Farnesol has an anti-obesity effect in high fat diet-induced obese mice and induces the development of beige adipocytes in human adipose tissue-derived mesenchymal stem cells [129]. This compound has potential anti-inflammatory and anti-cancer properties [130]. Cardioprotection by farnesol includes the role of the mevalonate pathway [131]. Nerolidol has a different pharmacological application in treating neurodegenerative diseases [132]. Nerolidol demonstrates anticholinesterase, antioxidant, anti-nociceptive, anti-inflammatory, and anxiolytic activities, thus it is considered as a promising phytochemical for the development of therapeutic drugs [133]. This compound has a neuroprotective effect against neuroinflammation, and oxidative stress induced by rotenone [61]. It exhibits anti-nociceptive and anti-inflammatory activity with the involvement of the GABAergic system and proinflammatory cytokines [134]. α-Bisabolol prevents neuronal damage and memory deficits through the reduction of proinflammatory markers induced by permanent focal cerebral ischemia in mice [135]. It reduces pro-inflammatory cytokine production and ameliorates skin inflammation [136]. This compound exhibits anti-nociceptive and anti-inflammatory activities in rodents [137,138]. Xanthorrhizol possesses antioxidant and anti-inflammatory activities in hippocampal neurons and primary cultured microglia [139]. It was shown anti-hyperglycemic and anti-inflammatory effects of xanthorrhizol in high-fat diet-induced obese mice [140]. This sesquiterpene demonstrates diverse pharmacological activities and anticancer properties [141]. It could be used as a pharmaceutical agent in disease management including cancer, infectious diseases, inflammatory process, metabolic syndrome, and platelet disorder. β-Elemene attenuates atherosclerosis in apolipoprotein E-deficient mice via restoring NO levels and alleviating oxidative stress [142]. It reduces the progression of atherosclerosis in rabbits [143]. β-Elemene has diverse mechanisms of influence on cancer cell interaction [144,145]. β-Caryophyllene has many effects on diseases of the nervous system. It exhibits a neuroprotective effect on cerebral ischemia-reperfusion injury via regulation of necroptotic neuronal death and inflammation [146]. β-Caryophyllene attenuates oxidative stress, neuroinflammation, glial activation, and salvages dopaminergic neurons in a rat model of Parkinson’s disease [147]. It showed a neuroprotective effect against dopaminergic neuron injury in a murine model of Parkinson’s disease induced by MPTP [148] and against cerebral ischemic injury [149]. β-Caryophyllene has antioxidant, anti-inflammatory, anticancer, cardioprotective, hepatoprotective, gastroprotective, nephroprotective, antimicrobial, and immune-modulatory activity [150]. Costunolide suppresses inflammatory angiogenic response in a subcutaneous murine sponge model [151] and ameliorates the inflammatory process associated with experimental pleurisy in mice [152]. Parthenolide has effects on neurological diseases, cancer, metabolism regulation and inflammation, inhibits the initiation of experimental autoimmune neuritis [153], relieves pain and promotes M2 microglia/macrophage polarization in a rat model of neuropathy [154], shows a hepatoprotective effect in a rat model of nonalcoholic fatty liver disease [155]. It acts as an NF-κB inhibitor, that ameliorates diabetes-induced behavioral deficit, neurotransmitter imbalance, and neuroinflammation in the type 2 diabetes rat model [156]. Parthenolide shows anti-inflammatory effects [157], inhibiting pro-inflammatory cytokine production and exhibiting protection of collagen-induced arthritis in a rat [158].

#### 3.2.9. Additional Activities

Farnesol interacts with thyroid hormone receptor (THR) β 1 and inhibits THR-mediated signaling in MCF-7 human breast cancer cells [159]. Local delivery of β-elemene improves locomotor functional recovery by alleviating endoplasmic reticulum stress and reducing neuronal apoptosis in rats with spinal cord injury [160]. β-Elemene influences α-tubulin polymerization in human hepatoma HepG2 cells [161] and supplies protection of endothelial cells, inhibition of neointimal hyperplasia in an injured artery [162]. β-Caryophyllene protects against alcoholic steatohepatitis by attenuating inflammation and metabolic dysregulation in mice [163]. This compound promotes osteoblastic mineralization and suppresses osteoclastogenesis and adipogenesis in mouse bone marrow cultures in vitro [164]. It modulates carbohydrate metabolism in streptozotocin-induced diabetic rats [165] and supplies gastric cytoprotection [166]. Xanthorrhizol demonstrates estrogenic activity [167] and has antibacterial activity against foodborne pathogens [168].

### 3.3. Diterpenes

**Figure 3:**
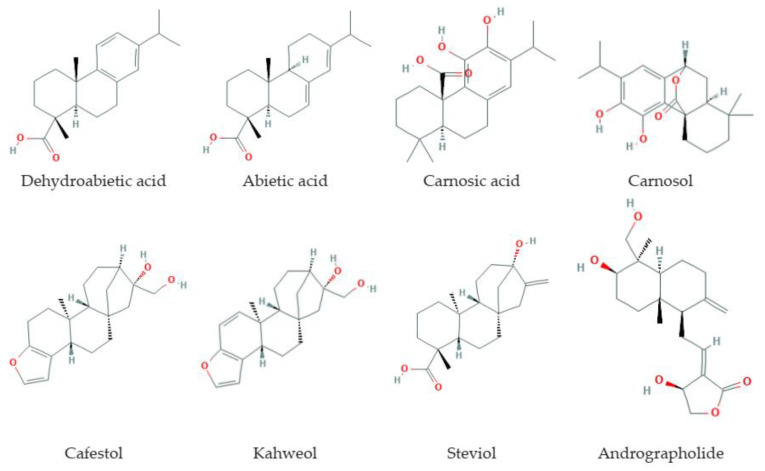
Some diterpenes that increase lifespan and delay age-related diseases.

Diterpenes (Figure 3) are C20-terpenoids constructed from four isoprene links, with the general molecular formula (C_10_H_16_)_2_. Diterpenes can have a linear, bi-, tri-, tetra-, and penta- or macrocyclic structure, depending on their skeletal core. In nature, diterpenes are usually found in the polyoxygenated form with keto- and hydroxyl groups. Diterpenes are the basis of biologically important compounds such as retinol, retinal, and phytol.

#### 3.3.1. Natural Sources

Diterpenes are widely distributed in plants of families such as *Lamiaceae, Verbenaceae, Celastraceae, Euphorbiaceae, Thymelaeaceae,* as well as in some genera such as *Rhododendron* and *Taxus*. Diterpenes and their derivatives are found in several food products (coffee, spices). Coffee diterpene alcohols, cafestol and kahweol, were isolated from green coffee beans (*Coffea arabica* L.). Carnosic acid (salvin) and carnosol are phenolic diterpenes present in spicy plants of the Lamiaceae family, such as rosemary (*Rosmarinus officinalis*), sage (*Salvia officinalis, Salvia pachyphylla*), oregano (*Origanum vulgare*) [169,170,171], basil (*Ocimum basilicum*), and thyme (*Thymus vulgaris*). Abietic and dehydroabietic acids are isolated from coniferous plants such as *Pinus densiflora, P. sylvestris, Abies grandis* [172], but also produced by representatives of several genera of cyanobacteria [173]. Andrographolide—bicyclic labdanum diterpenoid isolated from the stem and leaves of the medicinal plant *Andrographis paniculata* Nees. (*Acanthaceae*). Steviol is found in the leaves of *Stevia rebaudiana* Bertoni. (*Asteraceae*) [174]. Afidicoline is a tetracyclic diterpene antibiotic isolated from the fungus *Cephalosporum aphidicola*, also synthesized by *Nigrospora oryzae* [175].

#### 3.3.2. Lifespan Extension on Different Models

Effects of increased lifespan were observed for dehydroabietic acid and carnosol in experiments on *C. elegans*. Dehydroabietic acid has been shown to increase the lifespan in *C. elegans*, as well as prevent the accumulation of lipofuscin and the process of fibrosis [172]. Nematodes treated with carnosol and carnosic acid were characterized by an increase in average and maximum lifespan [176,177].

#### 3.3.3. Effects on Stress-Resistance

Diterpenes and diterpenoids have antioxidant activity. Carnosol and carnosic acid are inhibitors of lipid peroxidation, they prevent the oxidation of fatty acids, triglycerides, low-density lipoproteins in human aortic endothelial cells [178,179]. Under oxidative stress, nematodes treated with carnosol had a 21% increase in lifespan compared to controls, and under heat stress increased worm survival was higher by 9% [176]. The combined action of carnosic acid and carnosol against ROS and lipid radicals makes this diterpenoid tandem an effective antioxidant defense. In the Ames test, carnosol was found to have significant antioxidant and anti-mutagenic activity comparable to ascorbic acid [180]. In a micronucleus test, it was found that carnosol is even more effective than ascorbic acid in protection against gamma radiation [181]. Carnosol protects cells from eco-toxicants [182].

#### 3.3.4. Effects on Aging Biomarkers

Diterpenes affect the molecular, metabolic, and functional biomarkers of aging. The anti-aging effects of dehydroabietic acid are mediated by the activation of SIRT1 [172]. Diterpenes isolated from the leaf extract of *Croton tonkinensis* showed inhibitory activity on SIRT1 [183]. The effect of the cafestol is due to the inhibition of secretion of ICAM-1, MCP-1, and IL-8 and inhibition of phosphorylation of ERK and p38. The mechanism of action of cafestol is associated with the activation of HO-1 and SIRT1 [184].

#### 3.3.5. Toxicity and Side Effects

Diterpenes exhibit low toxicity. When using carnosic acid and carnosol, no visible damage to the body was observed, except for liver obesity in mice subjected to repeated administration of rosemary extract [185]. In animal and cell cultures, it has been shown that cafestol and kahweol show a wide range of biochemical effects, leading to a decrease in the genotoxicity of several carcinogens [186]. Steviol glycosides do not cause acute and subacute toxicity, allergic reactions, and they are not teratogenic, mutagenic, and carcinogenic substances. Their safety has been confirmed in numerous toxicological studies [187,188]. A few examples of side effects of some diterpenes are described. Therefore, high doses of taxol cause hair loss, bone marrow suppression, anemia, allergic reactions, muscle pain and diarrhea, heart problems, increased risk of infection, pneumonia, and neuropathy. The use of taxol during pregnancy is likely to cause birth defects in a fetus [189]. Abietic acid and dehydroabietic acid have pro-tumorigenesis cell transformation activity [190]. Dehydroabietic acid contributes to growth alterations and reproductive disturbances and affects liver energy metabolism in fishes [191,192]. In addition, diterpene abietane acids from pine needles and tips are abortifacient and toxic that was found in a study on cattle [193].

#### 3.3.6. Life Quality Effects

The effect of rosemary extract and its main components, rosmarinic and carnosic acids, on SOD1-G93A transgenic mice, which are models of amyotrophic lateral sclerosis, was studied. Rosemary diterpenes significantly delayed motor dysfunction, weakening the degeneration of motor neurons and increasing the lifespan of mice, improved clinical assessment, and reduced body weight loss [194]. Carnosic acid reduced the accumulation of epididymal fat in mice [195]. Carnosol slowed down the processes associated with aging, including age-related pigmentation and neurodegenerative diseases, while it did not affect fertility and fat deposition [176]. Kahweol and cafestol influenced the formation of bone tissue, inhibiting the differentiation of osteoclasts. Cafestol had an inhibitory effect on osteoclastogenesis and contributed to the differentiation of osteoblasts [196].

#### 3.3.7. Suppression of Pro-Aging or Activation of Anti-Aging Molecular Targets or Pathways

Diterpenes and diterpenoids affect several signaling pathways associated with aging, such as mTOR/AKT/PI3K, SIRT1, MAPK, NRF2, NF-κB, HSF1/HSP. Dehydroabietic acid shows itself to be an anti-aging agent that provides direct activation of SIRT1 [172]. Carnosic acid and carnosol induce the expression of heme oxygenase-1 (*HO-1*) gene and neuronal growth factors by inducing NRF2 and thus provide neuroprotective action. Carnosic acid has anti-inflammatory activity [197] and promotes neuronal differentiation [198]. Carnosol has anti-cancer, anti-inflammatory, and antioxidant effects mediated by modulating signaling cascades, including effects on molecules that regulate apoptosis (Bax/Bcl2), cell survival and proliferation (AKT/mTOR, MAPK), transcription factors, NF-κB, STAT3-6, and steroid androgen and estrogen receptors [199]. Kahweol enhanced the expression of *HO-1*, which provides neuroprotection from oxidative damage caused by 6-OHDA, and induced activation of PI3K and p38 [200]. Cafestol targets in endothelial cells are mitogen-activated protein kinases (MAPK), NRF2/HO-1 signaling pathway, and SIRT1 [184]. Cafestol weakened the action of intercellular adhesion molecules-1 (ICAM-1), monocyte chemoattractant protein (MSR-1), and the secretion of interleukin IL-8. Stevioside attenuated inflammation by reducing the expression of cytokine genes IL-6, TNF-α, and IL-1β in the mammary glands of mice infected with *S. aureus*, and inhibited the expression of cytokine genes by inactivating the MAPK, TLR2, and NF-κB pathways [201], suppressed proinflammatory cytokines [202,203,204]. Steviol and stevioside reduced the expression of IL-6, TNF-α, and IL-1β by inactivating NF-κB and activating Iκba in human colon carcinoma and THP-1 in vitro. These substances inhibit proinflammatory cytokines by increasing the level of IκBa [205,206]. Stevia leaf extract can effectively modulate the immune response and inhibit immunological disorders.

#### 3.3.8. Effects on Age-Related Diseases

Diterpenes and diterpenoids can be used in the prevention and complex treatment of cancer, neurodegenerative, cardiovascular, and metabolic disorders. Anticancer activity is indicated for taxol, abietic acid, andrographolide, kahweol and cafestol, steviol, and carnosol. Carnosic acid and carnosol exhibit antioxidant, anti-inflammatory, anticarcinogenic, and neuroprotective activity [199]. Dehydroabietic acid protects against ulcers and positively affects the state of the cardiovascular system [207,208]. Rosemary diterpenes—carnosic acid and carnosol—improve the redox status of the mammalian brain and modulate neuroinflammation, acting as neuroprotectors [209]. The inhibitory effect of carnosic acid on neurodegeneration in the CA1 region of the hippocampus in an experimental model of Alzheimer’s disease in rats was noted. Carnosic acid prevents obesity and glucose intolerance in mice, activates AKT and AMPKα signaling, enhances glucose uptake by skeletal muscle cells, reduces body weight and epididymal fat accumulation [195]. Kahweol has anti-inflammatory and anti-angiogenic effects. Cafestol helps reduce the risk of type II diabetes by stimulating insulin secretion and increasing glucose uptake in muscle cells [210,211]. Cafestol reduces the overall expression of inflammatory molecules in endothelial cells, inhibits the proliferation of vascular endothelial cells [184]. Cafestol palate and kahweol act against angiogenesis-dependent disorders [212]. Coffee extract with caffeine and cafestol are promising agents for controlling age-related neurodegenerative diseases due to their high bioavailability and low toxicity [213]. Coffee diterpenoids have a positive effect on model animals with symptoms of neurodegenerative diseases. Neuroprotective effects are shown in *Drosophila* models of Alzheimer’s disease and polyglutamine disease [214].

*Stevia rebaudiana* leaf extract has an antidiabetic effect by lowering blood glucose levels in patients with type 2 diabetes [215]. Isosteviol exhibits anti-inflammatory, antihypertensive activity, regulates blood lipids, is an immunomodulator, inhibits DNA polymerase and DNA topoisomerase, having anti-tumor, antioxidant, and anti-tuberculosis effects [187]. Stevioside and steviol affect β-cells and stimulate insulin secretion in mice and rats [216,217,218]. Steviol glycosides can lower blood pressure by modulating calcium and potassium channels, and repeated administration of stevioside in both normal and hypertensive mice led to an increase in glomerular filtration and renal blood flow [187,219]. Stevioside has an antihyperglycemic and hypotensive effect [217]. Clerodane derivatives have an NGF-potentiating effect and significantly increase NGF-mediated neurite growth in PC12 cells and show antiulcer activity [220]. Andrographolide demonstrates anti-inflammatory [221] and antibacterial activity [222], exhibits anti-allergic [223], antioxidant [224], and anti-cancer effects [225,226].

#### 3.3.9. Additional Activities

Diterpenes are diterpenoids and have antiviral, antibacterial, antiparasitic, and antifungal, antiprotozoal action. Some terpenoids are toxic to microorganisms and insects and play an important role in plant protection [220,227]. Extract of *Stevia* and its glycosides (for example, steviol), in addition to their value as sweeteners, have a therapeutic effect against cystic fibrosis. Carnosic acid and carnosol, isosteviol, andrographolide, dehydroabietic acid have several important protective properties, including anti-tuberculosis, antiseptic, and can be used in the treatment of colds, showing antibacterial and antiviral activity [171,187].

### 3.4. Triterpenes

**Figure 4:**
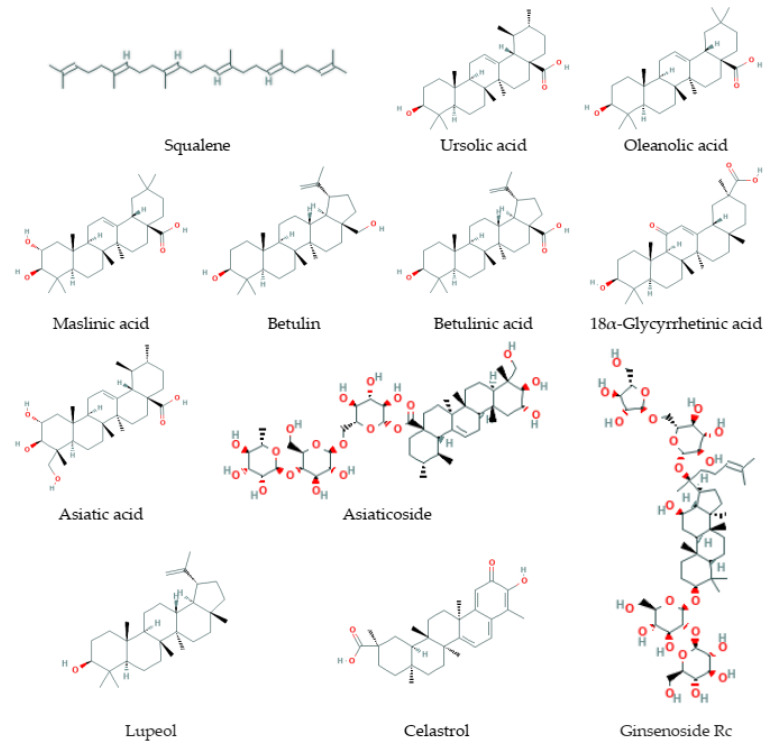
Some triterpenes that increase lifespan and delay age-related diseases.

Triterpenes (Figure 4) are derived from the C30 precursor, squalene, that consists of six C5 isoprene units with following cyclization and generation of downstream triterpenoid structures such as steroids, sterols, saponins (glycosides), and others [228,229]. Triterpenoids comprise more than 20,000 recognized molecules [230]. Triterpenes and triterpenoids play an important physiological role in a cell and an organism. These compounds are essential for cellular membrane formation and function and are a basis for the formation of signaling molecules such as steroid hormones and cognate receptors. Squalene is cyclized to lanosterol (a primary cholesterol and ergosterol precursor) and to cycloartenol (a precursor of β-sitosterol) [228]. Triterpenes and their metabolites demonstrate a wide range of biological activities against aging, inflammation, cancerogenesis, neurodegenerative, cardiovascular, metabolic diseases, viral, bacterial, and fungal infections [229,231,232,233,234,235].

#### 3.4.1. Natural Sources

Triterpenes and triterpenoids are biosynthesized by all known forms of life including bacteria, plants, fungus, and animals. Squalene is found both in many plants and animals including humans, it is an intermediate in the biosynthesis of phytosterol or cholesterol [236,237,238,239]. Triterpenes and triterpenoids with potential anti-aging activity are abundant in many plants. For example, oleanolic acid, ursolic acid, and betulinic acid are pentacyclic triterpene compounds contained in leaves, roots, and fruits of many plant species [240,241]. Some groups of triterpenoids are specific for plant species or families, particularly, ginsenosides (for *Panax ginseng*) [242] and limonoids (for citrus) [229]. Several triterpenes and triterpenoids were found in fungi, such as in fomitopsis (*Fomitopsis pinicola*), Poria (*Wolfiporia extensa*), and Reishi fungi (*Ganoderma lucidum*) [243,244,245]. Biological activity was also shown for synthetic triterpenoids [231,246,247,248], as well as there are methods for stimulation of triterpenoid production in medical plants [249].

#### 3.4.2. Lifespan Extension on Different Models

Ursolic and oleanolic acids were described as phytochemicals with significant pro-longevity action. The treatment with 25–50 µM ursolic acid and plant extracts reached with this compound increased the mean and maximum lifespan of *C. elegans* and *D. melanogaster* up to 30% [250,251,252,253]. Oleanolic acid at the 100–600 μM concentration extends the mean lifespan of nematodes by 10–20% [254]. *Stachys lavandulifolia* extracts that have betulin, betulinic acid, oleanolic acid, and ursolic acid among constituents sufficiently extend the *Drosophila* life [253].

Other potential geroprotectors are cucurbitane triterpenoids. Sea cucumber (*Holothuria scabra*) extracts containing triterpene glycosides increased the nematode’s mean lifespan by 5–8% [255]. Cucurbitane triterpenoids from *Momordica charantia* fruits extended the yeast replicative lifespan as well [256]. In addition, ginsenoside Rc increased the longevity of nematodes grown on media with different cholesterol content [257].

18α-glycyrrhetinic acid in wild-type nematodes and in a model of Alzheimer’s disease [258]. Some other triterpenoids (betulin, azadiradione, celastrol) demonstrated a pro-longevity action in model animals with symptoms of neurodegenerative diseases as well (Parkinson’s disease, Huntington’s disease, amyotrophic lateral sclerosis) [259,260,261].

#### 3.4.3. Effects on Stress-Resistance

Pro-longevity action of triterpenes and triterpenoids is associated with increased resistance to environmental stressors. Particularly, oleanolic acid [254], ursolic acid [250,251], as well as sea cucumber extract contained triterpene glycosides [255] improved the survival of nematode *C. elegans* in conditions of paraquat treatment and high temperatures. The cucurbitane glycoside increases yeast survival under oxidative stress and decreases ROS level [256]. This positive action can be mediated by the ROS scavenging activity and activation of antioxidant defense [251,254,262,263].

#### 3.4.4. Effects on Aging Biomarkers

Triterpenoids attenuated molecular, metabolomic, and functional aging biomarkers.

Studies carried on cultures of mammalian and human cells revealed that triterpenoids (for example, ginsenoside Rg1 and lupeol) inhibit cellular senescence markers, p53, p16, p21, Rb, SA-β-galactosidase, upregulated by senescence-induced treatments such as D-galactose or UV light [262,264]. Ursolic acid was shown to increase molecular anti-aging biomarkers, SIRT1, SIRT6, PGC-1β, and α-Klotho, in mouse hypothalamus [265].

Squalene improved the mitochondrial energy status in the liver of aged mice. This triterpene minimized alterations in the activity of tricarboxylic acid cycle enzymes and respiratory marker enzymes [266]. In rats, red ginseng extract with triterpene saponins restored nine biomarkers related to energy and lipid metabolism, that demonstrated the prevention of age-associated impairment of kidney function and amino acid metabolism disorders [267]. Additionally, the application of ginsenoside Rg1 in mice with D-galactose-induced damage improved oxidation-associated biomarkers, pro-inflammatory cytokine secretion, expression of senescence-associated proteins, and prevented the premature ovarian failure that indicated it possible activity against loss of reproductive functions due to aging-related pathologies [268].

The combination of Spirulina and glycyrrhizin, a saponin from licorice root, prevents cognitive dysfunction in aged rats with obesity. This effect was accompanied by a decrease of glucose, cholesterol, leptin levels in the serum, as well as a reduction in acetylcholinesterase activity in the hippocampus [269]. Maslinic acid supplementation provides maintaining muscular functions in elderly persons [270].

#### 3.4.5. Toxicity and Side Effects

Most triterpenes and triterpenoids that can be used as dietary components have low toxicity. For example, the subchronic toxicity and genotoxicity study of cycloastragenol did not reveal treatment-related mortality, sufficient side effects on different physiological parameters, or mutagenic action [271].

Undesirable effects of triterpenoids can be associated with its impact on reproductive functions. For example, consumption of ursolic acid by rats in an amount of 5 mg per kg of body weight suppresses spermatogenesis [272]. Additionally, triterpenes and triterpenoids can lead to gastrointestinal upsets [273].

#### 3.4.6. Life Quality Effects

Triterpenoids, for example, ursolic acid, and echinocystic acid, are described to improve learning ability and memory in mice [274,275]. Ginsenoside Rg3 and Rh2 from red ginseng roots have a calming effect [276]. Another positive action of triterpenoids is the stimulation and maintaining of physical activity. In rodents, treatment with ursolic acid or celastrol improves weight loss, condition of muscle tissue and muscle mass elevation, increases stamina, helps to maintain high motor performance for a longer time [260,277,278,279,280]. In *Drosophila*, ursolic acid increases in climbing activity. Furthermore, this triterpenoid led to flies’ microbiota changes that contribute to life- and healthspan extension [281]. Maslinic acid sufficiently improved mobility in the elderly. Particularly, its supplementation in the combination with moderate resistance training increased upper muscle mass and reduced knee pain, preventing disability [270].

#### 3.4.7. Suppression of Pro-Aging or Activation of Anti-Aging Molecular Targets or Pathways

Triterpenes and triterpenoids influence a set of pro- and anti-aging signaling pathways, particularly mTOR/AKT/PI3K, AMPK, SIRT1, MAPK, FOXO, NRF2, NF-κB, HSF1/HSPs pathways.

A set of triterpenoids demonstrate the ability to reduce phosphorylation levels of the mechanistic target of rapamycin (mTOR) and mTOR/AKT/PI3K pathway that lead to delay in aging and aging-related disorders (including cancer and neurodegeneration) [262,282,283,284,285,286,287,288]. Inhibition of mTOR mediates AMPK activation and autophagy induction, which play pro-longevity and anti-tumor role [283,284,285,286,287,288]. At the same time, ursolic acid in combination with leucine can stimulate myoblast differentiation and muscle mass increase through the induction of the mTOR pathway [278]. In addition, triterpenoid compounds exert anti-inflammatory action inhibiting NF-κB, COX-2, iNOS, TNF-α [260,289,290,291,292,293,294,295].

Triterpenoids and triterpenoid-containing extracts stimulate expression and enhance enzymatic activity of pro-longevity proteins SIRT1, SIRT6, PGC-1α, and JNK [251,265,275,281,296,297,298]. Furthermore, in silico, in vitro, and in vivo experiments demonstrated that ursolic acid directly binds to the outer surface of mammalian SIRT1 [297], as well as links with nematode JNK-1 ATP-binding site [250]. Oleanolic acid and sea cucumber extract modulated nuclear localization of FOXO/DAF-16 transcription factor and stimulated the activity of its downstream target genes *sod-3, hsp-16.2,* and *ctl-1* [254,255], as well as ursolic acid-activated SKN-1/NRF2 target genes *gcs-1* and *daf-9* [251] in *C. elegans*. Triterpenes and triterpenoids restored the normal expression of antioxidant enzymes (particularly, superoxide dismutase, catalase, glutathione peroxidase, glutathione reductase, glutathione-S-transferase—downstream members of FOXO and NRF2 signaling pathways) and the level of antioxidant molecules including glutathione, improved markers of oxidative stress in different tissues of rodent models [262,266,299,300,301]. At the same time, lifespan studies carried on *C. elegans* showed that pro-longevity effects of treatment with triterpenoids were abolished or decreased by mutations in *sir-2.1*, *jkk-1*, *jnk-1*, *sek-1, osr-1, daf-16, age-1, eat-2, skn-1* genes [250,251,254,255]. Additionally, triterpenoids were found to activate heat shock response (including HSF1 and HSP70 upregulation) and unfolded protein response [244,259,260].

#### 3.4.8. Effects on Age-Related Diseases

Triterpenes and triterpenoids can be used in the prevention and complex therapy of cancers, neurodegenerative, cardiovascular, and metabolic disorders.

At the present time, there are a number of pieces of evidence that demonstrated anti-cancer properties of the pentacyclic triterpenoids of oleanane-, ursane, lupane, and friedelane types (including oleanolic, ursolic, betulinic, 18α-glycyrrhetinic, asiatic acids, celastrol, lupeol, among others) [231,302]. These compounds suppress tumor growth, reduce survival, and induce apoptosis of different types of cancer cells, including skin, breast, colon, prostate tumor cells, and others [290,303,304]. However, treatment with pentacyclic triterpenoids at effective anti-cancer concentrations had a toxic effect on normal cells in some cases [304]. A promising anti-cancer potential has triterpenoids extracted from formopsis, poria, and Reishi fungi. In vitro studies demonstrated their effects in murine Sarcoma cancer cell line and human leukemia, liver cancer, esophageal cancer, pancreatic cancer, prostate cancer cell lines [243,244,305]. In vivo study also revealed anti-tumor effects and demonstrated the survival improvement of tumor-bearing mice [243,244]. Furthermore, fungus triterpenoids had a little toxic impact on normal cells and tissues.

In addition, a number of semisynthetic derivatives of pentacyclic triterpenoids have been synthesized. Some of them were shown to have improved therapeutic activity, pharmacokinetic properties, and less toxicity for normal cells and tissues compared with parent compounds [231,247,248]. For the enhancing of bioavailability and therapeutic efficiency, delivery nanosystems are developed for triterpenoids such as ursolic, oleanolic, and betulinic acids [306,307]. Squalene can be used as an adjuvant in cancer chemotherapy and protect normal tissues against the toxic influence of some anti-cancer agents [308,309,310]. Squalene-based nanoparticles with cisplatin, doxorubicin, or paclitaxel were developed as prodrugs for targeted chemotherapy [311,312,313]. This approach can be also used for the treatment of other disorders. Particularly, squalene-adenosine nanoparticles have a high potential for the neuroprotection in stroke and spinal cord injury [314].

Triterpenes and triterpenoids can be used for the treatment of neurodegenerative disorders such as Alzheimer’s disease and Parkinson’s disease, and for the prevention of aging-dependent cognitive impairment. These compounds improve cognitive functions, learning and memory abilities, prevent synaptic plasticity dysfunction, β-amyloid peptide (Aβ) deposition and toxicity, suppress senescence and death of neural stem and neuronal cells, decrease inflammation and oxidative stress, correct metabolic and hormonal imbalance in models of accelerated senescence and neurodegeneration in vivo and in vitro [258,262,301,315,316,317,318,319].

Squalene and a number of triterpenoids can be used as cardioprotector agents due to their ability to reduce levels of low-density lipoprotein cholesterol (with a rise in the level of high-density lipoprotein cholesterol) and triglycerides, antioxidant and anti-inflammatory properties [289,320,321,322,323,324,325]. Triterpene compounds prevent structural changes in the myocardium, development of cardiovascular pathologies, and support normal cardiac function. In studies on rats, the protection action of squalene and triterpenoids against myocardial infarction, blood pressure increase, ischemia-reperfusion injury, chronic heart failure was found [320,321,322,323].

A range of pentacyclic triterpenoids contribute to metabolic syndrome through the regulation of proteins and signaling pathways involved in adipogenesis, lipolysis, fatty acid oxidation, insulin resistance, mitochondria biogenesis, gluconeogenesis, oxidative stress, and inflammation [233]. Squalene and triterpenoids can reverse hyperglycemia status, which is useful for the treatment of such metabolic diseases as diabetes mellitus and obesity. These compounds decrease levels of glucose in blood and triglycerides in the liver, stimulate insulin production, induce enzymatic and non-enzymatic antioxidant activities in model animals with diabetic symptoms, and diet with high fat or sucrose [326,327]. Triterpenoids can be applied to mitigate obesity and hyperlipidemia. These compounds lead to the destruction of lipids in adipocytes, inhibition of preadipocyte differentiation, and reduction of body fat content [251,321,328,329].

In addition, oleanolic acid has bone anti-resorption activity in aged female rats and can be applied in osteoporosis prevention [330]. Due to anti-inflammatory activity, triterpenoids, such as maslinic acid, can prevent related diseases, particularly arthritis [294,295]. Squalene and ginsenosides can be used for the protection against skin photoaging [242,273]. Their capacity was demonstrated in clinical trials and in vitro studies. Lupeol improved the selenite-induced cataract in rats and decreased the oxidative stress in eye tissues [300].

#### 3.4.9. Additional Activities

Triterpenes and triterpenoids have antiviral, antibacterial, antifungal, antiparasitic action [230,331,332,333,334,335,336,337]. Furthermore, triterpenoids can be used as immunomodulator agents [246].

### 3.5. Tetraterpenes or Carotenoids

**Figure 5:**
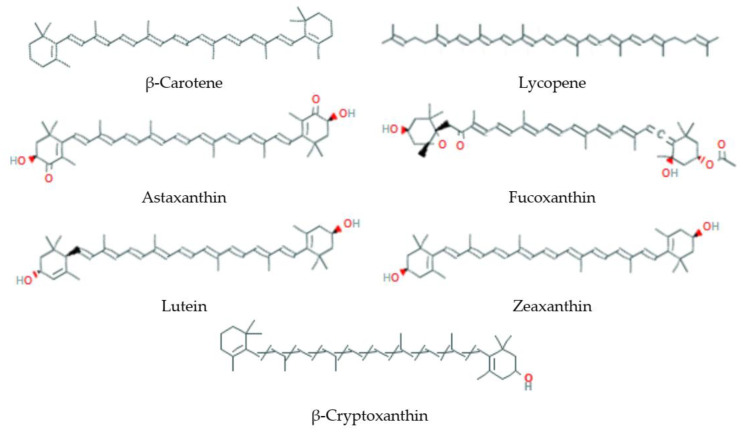
Some carotenoids that increase lifespan and delay age-related diseases.

Carotenoids (Figure 5) are pigments that contain in their structure the C40 hydrocarbon backbone [338]. Depending on the presence of oxygen in the structure, carotenoids are divided into (i) unoxygenated carotenoids (β-carotene, α-carotene, lycopene) and (ii) oxygenated xanthophylls (astaxanthin, fucoxanthin, lutein). Furthermore, they can be divided into two groups depending on their ability to possess provitamin A activity. In nature, carotenoids play two important roles in the photosynthesis process by harvesting light and protecting an organism from excessive light exposure, which leads to ROS formation [339].

#### 3.5.1. Natural Sources

Around 600 different carotenoids are known, 40 of which people consume with food [340]. In European countries, the most abundant carotenoids in food are lutein and β-carotene [341]. Lutein can be found in different green leafy vegetables like kale, spinach, asparagus, parsley, broccoli, zucchini, and lettuce [342]. β-Carotene, a precursor of Vitamin A, is the most abundant carotenoid in nature and contains in a variety of fruits and vegetables like spinach, pepper, lettuce, kale, and apricot [342].

#### 3.5.2. Lifespan Extension on Different Models

Even though the influence of different carotenoid-containing extracts on lifespan parameters of model organisms is excessively studied [343,344,345], the data with pure carotenoids are limited. It was shown that β-carotene increased the median lifespan of *D. melanogaster* females up to 37% and the age of 90% mortality up to 11% [346]. In *D. melanogaster* males the effects of β-carotene were not stable. In another study, synthetic all-trans-carotene (100 μM) had no effects on the lifespan parameters of both *Drosophila* males and females [344]. This compound did not have statistically significant effects on the lifespan parameter of *C. elegans* at the concentrartions of 0.3–10 μM [346]. The statistically significant effects were also absent in experiments with mice [347].

Carotene lycopene had no effects on *C. elegans* lifespan parameters [348]. At the same time, pure synthesized lycopene at a dose of 7.5 ppm increased the mean lifespan of both *D. melanogaster* males and females by 4.8–8.1% as well as the maximum lifespan of females by 4.8% [345]. The positive effects were sex-dependent and more pronounced in females. They were accompanied by an increased SOD level.

Xanthophyll fucoxanthin in the concentration of 5 μM enhanced the median and maximum lifespan parameters in wild-type *C. elegans* species by 14% and 24%, respectively [346]. The addition of fucoxanthin also had positive effects on the longevity of *D. melanogaster*. In one study, it was shown that the compound increased the median lifespan of *D. melanogaster* females up to 49% and the age of 90% mortality up to 27% [346]. In *D. melanogaster* males the effects of fucoxanthin were not stable. In another study, the positive effects of 1 μM xanthophyll on *D. melanogaster* lifespan parameters were observed in both sexes [349].

Another xanthophyll astaxanthin (30 μM) increased the chronological lifespan of wild-type *S. cerevisiae* strains [350]. The positive effects were also observed on antioxidant (*sod1*Δ, *sod2*Δ, *tsa1*Δ, *cta1*Δ) and anti-apoptotic (pep4Δ, fis1Δ) deficient *S. cerevisiae* strains. Astaxanthin in concentrations 0.1–1 mM also increased the mean lifespan of wild-type *C. elegans* up to 31% [351]. It was shown that the positive effects on *C. elegans* lifespan parameters were observed only if the compound was fed to animals throughout their entire life or only at adult stage [348]. No effects were found, when astaxanthin has been fed to worms only from the larval L1 stage until adulthood. Astaxanthin increased longevity of the mealworm beetle *Tenebrio molitor* [352].

The addition of lutein in amounts of 0.03 and 0.1 mg to ml diet increased the mean and maximum lifespan of *D. melanogaster* males up to 11.4% and 16%, respectively [353]. The positive effects were accompanied by the upregulation of expression levels of a few antioxidant enzyme genes (*Sod1*, *Sod2*, *Cat*). The effects on flies’ females were not studied. Zeaxanthin had no effects on *C. elegans* lifespan parameters [348]. 

#### 3.5.3. Effects on Stress-Resistance

β-Carotene increased resistance of *Drosophila* females to oxidative stress but had negative effects on the resistance of *Drosophila* males to heat shock [346]. The effects of fucoxanthin on different stress conditions were also controversial [346,349].

Lutein (0.1 mg/mL diet) enhanced resistance of *D. melanogaster* males to oxidative stress generated by the addition of paraquat and H_2_O_2_ [353].

A 2-h pretreatment with astaxanthin (30 μM) decreased sensitivity of *S. cerevisiae* antioxidant deficient strains (*sod1*Δ, *sod2*Δ, *tsa1*Δ, *cta1*Δ, *ctt1*Δ) to oxidative stress induced by H_2_O_2_ [350]. The positive effect was accompanied by the decreased levels of ROS and lipid peroxidation as well as an increased level of glutathione (GSH) in all studied antioxidant deficient strains. In strains *tsa1*Δ, *cta1*Δ, and *ctt1*Δ increased SOD enzyme activity was observed. Furthermore, astaxanthin decreased apoptotic cell death under acetic acid and H_2_O_2_ treatments in mutant strains, which exhibit increased apoptosis due to the lack of Fis1p or Pep4p proteins. Astaxanthin reduced growth defects in both antioxidant and anti-apoptotic deficient strains induced by H_2_O_2_ or acetic acid treatment. Astaxanthin decreased resistance of the mealworm beetle to a bacterial infection induced by using its two known entomopathogenic bacterial pathogens *Bacillus cereus* and *Bacillus thuringiensis* [352].

#### 3.5.4. Effects on Aging Biomarkers

Fucoxanthin exerts protective effects in human fibroblasts cellular senescence [354]. The addition of fucoxanthin to *D. melanogaster* decreased the proportion of the male flies with increased intestinal permeability (Smurfs), a known-biomarker of aging in a number of species [355], and had no effects on this parameter in females [349].

Lutein (0.03–0.1 mg/mL) reduced the level of malondialdehyde, an end-product of lipid peroxidation, and a potential biomarker of aging [356], in *D. melanogaster* males [353]. The same effects were observed after the addition of pure synthesized lycopene [345].

One of the aging-related behavioral changes observed in organisms is a breakdown of sleep:wake cycles [357,358]. Fucoxanthin decreased sleep disturbance in old *Drosophila* females at night but decreased this parameter in younger flies [349]. In contrast to the previously mentioned positive effects of carotenoids on aging biomarkers, astaxanthin caused an immune depressive effect in the mealworm beetle [352].

#### 3.5.5. Toxicity and Side Effects

Carotenoids are well-known antioxidants, however, under certain conditions, they may display pro-oxidant properties too. The key factors in explaining the dual role of carotenoids are oxygen pressure and carotenoids’ concentration [359]. Experimental data show that β-carotene can increase the possibility of cancer development in smoking men [360]. The analysis of randomized trials showed that β-carotene could also enhance mortality both in healthy people and people with various diseases [361]. Excessive consumption of β-carotene can lead to carotenosis, e.g., to skin orange discoloration [362].

However, in general, carotenoids are regarded as non-toxic compounds. They are commonly used as food colorants [362,363]. Nevertheless, the questions regarding which compounds can be used in which food, the compound’s levels, their sources, and purity are subject to legislation regulation in different countries [363].

#### 3.5.6. Life Quality Effects

Both β-carotene (0.3–1 μM) and fucoxanthin (0.3–1 μM) increased locomotor activity in males but had no effects on this parameter in females [346,349]. Synthetic all-trans-carotene (100 μM) improved mobility of eight weeks of age females in a negative geotaxis test [344].

In *D. melanogaster* females, no statistically significant effects of β-carotene (0.3–1 μM) on the fecundity parameters like egg-laying and pupae development from eggs were observed [346]. Pure synthesized lycopene increased the reproductive activity of *D. melanogaster* females by enhancing the amount of F1 generation and the sexual capacity (mating rate and mating duration time) [345]. The effects of fucoxanthin in tested 0.3–1 μM concentrations on *D. melanogaster* females’ egg-laying were controversial. The compound stimulated egg-laying by 30–48% in young female flies on the first experimental week, but in most cases decreased this parameter by 2–77% on other weeks. Fucoxanthin enhanced the number of pupae development from eggs in the concentration of 1 μM [346,349]. Supplementation with astaxanthin increased larval development time of the mealworm beetle *Tenebrio molitor* [352].

#### 3.5.7. Suppression of Pro-Aging or Activation of Anti-Aging Molecular Targets or Pathways

Carotenoids possess direct scavenging properties due to the presence of conjugated double bonds in their structure [364]. The compounds are also able to modify activities of different anti-aging molecular targets or pathways. For example, it was found that carotenoids can induce antioxidant defense mechanisms in the cell by activating the transcription factor NRF2 [365]. Lycopene and fucoxanthin were reported to increase the translocation of NRF2 to the nuclei and induce expression of phase II enzymes via activation of the ARE transcription system [366,367]. The effects of other tested carotenoids β-carotene, astaxanthin, and phytoene were less expressed [366].

Carotenoids inactivate NF-κB, which triggers the transcription of inflammatory cytokines [365]. For example, β-carotene suppressed activation of NF-κB pathway in LPS-pretreated RAW264.7 cells and peritoneal macrophages by decreasing translocation of NF-κB p65 subunit to the nuclei and phosphorylation of an NF-κB inhibitor protein IκB, which led to a suppression of IκB degradation. The β-carotene treatment also reduced the expression of inflammatory molecules [368,369]. 

Carotenoids modulate the MAPK activity. For example, β-carotene may activate or inactivate JNK and p38 depending on the concentration used [370]. Fucoxanthin activated JNK in cancer cells, which resulted in cell cycle arrest on G1-phase [371,372].

The fucoxanthin induced cell cycle arrest was also associated with increased GADD45 expression [371,372]. *GADD45* overexpression led to increased *D. melanogaster* lifespan probably due to the resulting increase in the efficiency of detection and repair of spontaneous DNA damage [373].

The effects of carotenoids on the insulin/IGF-1 signaling pathway, a well-known longevity regulator, are controversial. For example, Yazaki et al. proposed that the insulin/IGF-1 signaling pathway might be one of the mechanisms of astaxanthin positive action on *C. elegans* lifespan [351]. They observed that in the *age-1* mutant the positive effects of astaxanthin were less expressed than in wild-type worms. In *daf-16* null mutants, no significant results on lifespan were found. At the same time, astaxanthin increased nuclear localization of the DAF-16 transcription factor as well as increased expression levels of DAF-16 target genes. The effects of astaxanthin were also associated with decreased ROS production in mitochondria. However, in another study, it was shown that the effects of astaxanthin on *C. elegans* were independent of the insulin/IGF-1 signaling pathway as the positive effects of the compound were also present in experiments with *daf-2(e1370)* and *daf-16(mu86)* strains [348]. Experiments with *eat-2 (ad1116)* mutants revealed that they were also independent of the dietary restriction mechanism. The main mechanism of astaxanthin proposed by the authors is that the compound affects biogenesis and activity of the mitochondrial respiratory chain complex III, which possibly results in decreased mtROS production due to the decreased electron leakage [348].

#### 3.5.8. Effects on Age-Related Diseases

The possible role of carotenoids in inhibiting the development of various types of cancer is being actively discussed [374,375]. The anticancer activity was noted for several carotenoids (β-carotene, α-carotene, lycopene, lutein, zeaxanthin, β-cryptoxanthin, fucoxanthin, canthaxanthin, and astaxanthin). However, the negative effects were also reported [376].

There is evidence of neuroprotective effects of carotenoids. For example, it has been shown that carotenoid fucoxanthin decreased the pro-inflammatory response of microglia, reducing the production of neurotoxic mediators such as nitric oxide (NO) and pro-inflammatory cytokines (TNF-α, IL-6, IL-1β) [377]. The effects were associated with inhibition of the activation of mitogen-activated protein kinases (ERK, JNK, p38). In addition, fucoxanthin modulates the response of microglia to oxidative stress, activating the expression of antioxidant defense genes and reducing the level of free radicals in the cell.

The anti-obesity effects were reported for β-carotene, astaxanthin, β-cryptoxanthin, fucoxanthin, zeaxanthin, and lycopene in experiments using mice [378]. Carotenoids were able to inhibit adipogenesis and activate adipocyte browning and lipid catabolism. The direct influence of carotenoids on the brain function as a possible mechanism of carotenoids’ anti-obesity properties is being discussed. The available studies on humans, even though mostly dealing with mixtures of different carotenoids, confirm the beneficial effects of carotenoids on obesity.

Carotenoids prevent age-related cognitive decline. A few studies showed that the higher serum levels of lycopene, lutein, zeaxanthin, and β-carotene are associated with better cognitive performance in old people [379,380,381,382]. There is also evidence of beneficial effects of β-carotene in people with Alzheimer’s and Parkinson’s disease [383,384].

Carotenoids have potentially positive effects on cardiovascular health. Epidemiological studies show that there is a correlation between risk of cardiovascular diseases and atherosclerosis and concentration of carotenoids in dietary intake, plasma or serum, adipose tissue [385]. In clinical trials, the positive effects are also observed. For example, the supplementation with lycopene in form of cooked tomatoes, tomato extracts, or tomato juice lowered blood pressure, decreased concentration of lipid peroxidation products, reduced levels of total and LDL cholesterol, increased levels of antioxidant enzymes and resistance of LDL cholesterol to oxidation [385,386]. It also caused a decrease in levels of C-reactive protein and adhesion molecules (VCAM-1 and ICAM).

The positive correlation between carotenoids’ dietary intake or serum concentrations of carotenoids like β-carotene, α-carotene, β-cryptoxanthin and risk of type 2 diabetes was revealed in a number of studies [387,388,389,390,391]. However, contradictory data are also available [387,388].

Lutein and zeaxanthin are the most studied carotenoids in relation to eye health as they both are abundant in the macula, where they function as filters of blue light and provide protection against light-induced damage [392]. The potential beneficial effects of lutein and zeaxanthin on several eye diseases including age-related macular degeneration, cataracts, retinitis pigmentosa, retinopathy of prematurity, and diabetic retinopathy are being discussed [393,394,395]. The carotenoids supplementation had been reported to improve visual acuity, contrast sensitivity, and macular pigment optical density levels. It is reported that carotenoids like β-cryptoxanthin and lycopene have beneficial effects on bone health by activating osteoclasts and/or depressing the work of osteoblasts [396,397].

### 3.6. Polyterpenes

Polyterpenes are composed by many isoprenyl groups in the side chain ((C_5_H_8_)*_n_*, where *n* > 8). Some hardwoods produce polyterpenes—rubber and gutta (gutta-percha). Natural rubber consists of polyisoprene, in which double bonds are in the cis-conformation. Some plants produce polyisoprene, in which the double bond is in trans-conformation, this is gutta-percha. This class of terpenoids does not have geroprotectors properties, but these compounds are widely used materials because they have low toxicity [398,399,400,401].

### 3.7. Norisoprenoids

Norisoprenoids have 13 carbon atoms and are found in *Vitis vinifera* grape leaves. These include 3-oxo-α-ionol presented in the Muscat of Alexandria variety and derivatives of 7,8-dihydroionones, such as megastigman-3,9-diol and 3-oxo-7,8-dihydro-α-ionol, found in the Shiraz variety, also β-damascenone, 3-hydroxy-β-damascenone, 1,1,6-trimethyl-1,2-dihydronaphthalene in the Merlot grape [402,403,404,405,406]. This class of terpenoids does not have a geroprotectors property as well. Norisoprenoids add flavor to wine. Nowadays, clinical uses are being developed. For example, a method of sunburn prevention by β-damascenone in Skh-1 mice was studied [405].

### 3.8. Sesterterpenes

These terpenes, having 25 carbon atoms and five units of isoprene, are rarely found, and insufficiently explored. An example of sesterterpenes is geranylpharnesol. Geranylpharnesol has been shown to induce the differentiation of mouse myeloid leukemia M1 cells into macrophage-like cells. It was also found that geranylpharnesol can inhibit DNA and RNA synthesis by specifically inhibiting rRNA synthesis [407]. Studies on the tumor specificity of geranylpharnesol have shown that it has cytotoxicity against certain cultured human tumor cells [408].

### 3.9. Sesquarterpenes

Sesquarterpenes are composed of seven isoprene units and have the molecular formula C_35_H_56_. They are usually synthesized only in microorganisms. Sesquiterpenes are insufficiently explored. Examples of sesquarterpenoids are ferrugicadiol and tetraprenylcurcumen. It has been shown that ferrugicadiol from *Calocedrus macrolepis* var. *Formosana* is cytotoxic to human epidermoid carcinoma cells (KB cells) [409].

## 4. Possible Prospect of Terpene using as Anti-Aging Drugs

Due to their numbers and diversity, terpenoids offer much potential in an array of industrial and medicinal applications among all the secondary metabolites of plants [410]. Although most of the terpenes have not yet been fully investigated, they are known to have a wide range of medicinal applications among which are antiplasmodial, antiviral, anticancer, antidiabetic, and antidepressant activities [3,12,411]. Here we showed that most classes of terpenoids have representatives with the explicit geroprotective properties as antioxidants and inducers of the expression of cytoprotective mechanisms. The greatest compliance with the primary and secondary criteria of geroprotectors we found for the following terpenoids: limonene (monoterpene), β-caryophyllene (sesquiterpene), dehydroabietic acid, carnosol, carnosic acid (diterpenes), squalene, ursolic acid, oleanolic acid, maslinic acid, 18α-glycyrrhetinic acid, asiaticoside, ginsenosides (triterpenes), β-carotene, lycopene, fucoxanthin, astaxanthin, lutein, zeaxanthin, β-cryptoxanthin (tetraterpenes/carotenoids) (Table 1). In addition, terpenoids with known medicinal applications potentially could be repurposed to combat aging and prevent age-related conditions in humans [412].

Along with their structural diversity and a broad spectrum of physiological activities, terpenes also allow for flexibility in the route of administration [411]. Possible routes of administration for terpenoids may include, but are not limited to, cutaneous (administration to the skin as anti-aging cream components [507]), oral (consumption as pharmacological substances, nutritional supplements, and bioactive food components [411]), and respiratory (inhaling the essential oils of aromatic plants or natural forest atmosphere [3,12,508]).

The experimental validation of the anti-aging activity of terpenes in humans is one of the major challenges to revealing new geroprotectors. According to a recent review, three major approaches are applicable to test anti-aging interventions in humans and accelerate their widespread use to improve human aging [509]. These approaches include testing of longevity interventions in the context of age-related disease or process indications; investigating the preventing effect of anti-aging interventions on multiple chronic diseases simultaneously; using non-invasive or minimally invasive strategies to measure biological age [509]. All these promising approaches can be applied to prove the activity of terpenoids as potential geroprotectors.

## 5. Conclusions

The unprecedented increase in the average human lifespan is one of the greatest accomplishments of the past century [510,511]. Life expectancy will continue to increase for the foreseeable future, which in combination with a rapid decline in human fertility will lead to population aging [510,512]. At the same time, healthspan (healthy, disease-free lifespan) has not increased as much as lifespan [511,513]. Since aging is the predominant risk factor for most chronic diseases, fragility, and disability in the elderly, population aging has become one of the main global challenges [511,514,515]. The development and implementation of effective geroprotective interventions can contribute to healthspan increasing and prevention or amelioration of age-related pathologies [6,509].

In this review, we showed that most classes of terpenoids have representatives with the potential geroprotective properties (Table 1). Thus, terpenoids are underestimated in their potential activities in terms of criteria of geroprotectors. We suggest that these compounds have a great prospect to become a new class of anti-aging drugs.

## Figures and Tables

**Table 1:** Geroprotective potential of some terpenes and terpenoids.

Compound (Molecular Formula)	Compliance with Criteria of Geroprotectors *									References
	Primary Criteria					Secondary Criteria				
	1	2	3	4	5	6	7	8	9	
Monoterpenes (C_10_H_16_)										
Limonene	+	+	-	-	+	+	+	+	+	[22,23,28,35,37,38,39,40,41,44,46,47,48,413,414,415,416,417]
Sesquiterpenes (C_15_H_24_)										
β-Caryophyllene	+	+	+	+	+	+	0	+	+	[58,67,79,85,86,93,96,105,147,150,164,418,419,420,421,422,423,424]
Diterpenes ((C_10_H_16_)_2_)										
Dehydroabietic acid	+	+	-	-	-	+	0	+	0	[172,190,191,192,193,207,208,425,426,427]
Carnosol	+	+	+	+	+	+	0	+	+	[176,181,185,186,199,209,428,429,430,431,432]
Carnosic acid	+	+	+	+	+	+	0	+	+	[177,185,194,195,197,209,429,433,434,435,436,437]
Triterpenes ((C_10_H_16_)_3_)										
Squalene	0	+	0	+	0	+	0	+	0	[236,266,273,308,309,310,314,320,321,324,326]
–Ursolic acid	+	+	+	-	+	+	+	+	+	[231,250,251,265,281,297,298,302,438,439,440,441,442,443]
Oleanolic acid	+	0	+	-	+	+	0	+	+	[231,254,302,322,323,330,444,445,446,447,448]
Maslinic acid	0	+	+	+	+	+	0	+	0	[231,270,294,295,325,449]
18α-Glycyrrhetinic acid	+	0	-	-	+	+	0	+	0	[231,258,439,450,451,452,453,454,455,456]
Asiaticoside	0	+	+	0	+	+	0	+	0	[231,301,302,457,458,459,460,461,462,463,464,465]
Ginsenosides	+	+	+	-	+	+	0	+	+	[257,262,268,466,467,468,469,470,471,472,473,474,475,476,477,478]
Tetraterpenes/carotenoids ((C_10_H_16_)_4_)										
β-Carotene	-	+	+	-	+	+	-	+	-	[344,346,347,360,361,362,365,370,376,378,383,384,479,480,481,482,483,484]
Lycopene	+	+	+	+	+	+	-	+	0	[345,348,362,365,366,376,378,479,484,485,486,487,488,489]
Fucoxanthin	+	+	+	+	+	+	+	+	-	[346,349,362,365,367,371,372,377,378,490]
Astaxanthin	+	+	+	-	+	+	+	+	+	[326,350,351,352,362,365,376,378,491,492,493,494,495]
Lutein	+	+	+	+	+	+	0	+	+	[353,362,365,376,393,394,395,481,484,496,497,498,499,500]
Zeaxanthin	-	+	+	+	+	+	0	+	0	[348,362,365,376,378,393,394,395,481,484,498,499,500,501]
β-Cryptoxanthin	0	+	+	0	+	+	0	+	0	[362,376,378,479,483,484,499,502,503,504,505,506]

* Primary criteria: 1. The life extension in wild-type animal models, 2. Improvement of aging biomarkers, 3. Low toxicity, 4. Minimal side effects, 5. Improvement of the quality of life; Secondary criteria: 6. Evolutionarily conserved mechanisms, 7. Reproducibility on different models, 8. Prevention of age-associated diseases, 9. Increased stress-resistance. + Compliance with criteria; - Not compliance with criteria; 0 Not investigated.
